# Linking the heart and brain in type 2 diabetes: association between global longitudinal strain and cognitive function

**DOI:** 10.3389/fendo.2025.1689792

**Published:** 2025-10-08

**Authors:** Federica Barutta, Alessandro Andreis, Martina Bollati, Arianna Ferro, Stefania Bellini, Giulia Gioiello, Giulio Mengozzi, Matteo Bellettini, Gaetano M. De Ferrari, Gianluca Alunni, Fabio Broglio, Guglielmo Beccuti, Gabriella Gruden

**Affiliations:** ^1^ Department of Medical Sciences, University of Turin, Turin, Italy; ^2^ Advanced Cardiovascular Echocardiography Unit, Cardiovascular and Thoracic Department, Città della Salute e della Scienza di Torino University Hospital, Turin, Italy; ^3^ Division of Cardiology, Città della Salute e della Scienza di Torino, University Hospital, Turin, Italy

**Keywords:** global longitudinal strain, type 2 diabetes, cognitive performance, education, left ventricular ejection fraction (EF)

## Abstract

**Introduction:**

Cognitive impairment is a frequent complication of type 2 diabetes (T2DM). Global longitudinal strain (GLS), an echocardiographic marker of subclinical left ventricular (LV) systolic dysfunction, has been associated with adverse cardiovascular outcomes in T2DM. However, its relationship with cognitive performance remains unexplored. The aim was to investigate the association between GLS and cognitive function in patients with T2DM.

**Methods:**

We prospectively enrolled 234 T2DM patients without hemodynamically significant carotid stenosis, history of stroke or severe hypoglycemia. Cognitive function was assessed using the Repeatable Battery for the Assessment of Neuropsychological Status (RBANS) and GLS measured via speckle-tracking echocardiography. Multivariable linear regression models were used to evaluate associations between GLS and RBANS scores. Sensitivity analyses excluded individuals with coronary heart disease (CHD), atrial fibrillation (AF), or LV ejection fraction (LVEF) <50%.

**Results:**

The mean RBANS total score was 96.7 ± 17.1; 19.7% of participants scored <80, indicating borderline/impaired cognition. Mean GLS was −19.23 ± 2.59%, with 29.1% of patients showing subclinical LV dysfunction (GLS ≥ −18%). Unlike LVEF, impaired GLS (≥ −18%) was associated with lower RBANS total scores. This association remained significant after excluding individuals with CHD, AF, or LVEF<50%, and after adjusting for age, sex, education, lifestyle factors, metabolic and hemodynamic parameters. Educational attainment modified the association, with stronger GLS-cognition links in participants with lower education. The relationship was unaffected by adjustment for markers of inflammation and endothelial dysfunction.

**Conclusions:**

In patients with T2DM, impaired GLS is independently associated with reduced cognitive performance, even in patients with normal LVEF.

## Introduction

Cognitive impairment is a well-recognized yet often underdiagnosed complication of Type 2 diabetes (T2DM) and ranges from subtle neurocognitive decrements to overt dementia (1, 42). Up to 45% of individuals with T2DM may exhibit signs of mild cognitive impairment, particularly affecting memory, executive function, and attention (2, 3). These deficits can significantly compromise quality of life and functional independence (4), yet they often remain undetected in routine clinical practice.

Cardiac dysfunction, particularly heart failure (HF), has also been independently associated with cognitive impairment and an increased risk of dementia (5). In the general population, reduced left ventricular ejection fraction (LVEF), particularly below 30%, has been linked to lower cognitive performance (6). Patients with HF frequently show impairments in attention, memory, visuospatial processing, and executive function (7), and cognitive dysfunction in this setting is associated with worse clinical outcomes, including higher rates of hospitalization and mortality (8–13).

Global Longitudinal Strain (GLS), measured through speckle-tracking echocardiography, has recently emerged as a more sensitive marker of early subclinical left ventricular systolic dysfunction than LVEF. GLS can detect early myocardial impairment even in individuals with preserved ejection fraction (14; 15). Importantly, GLS is often impaired in patients with T2DM and has been independently associated with increased risks of hospitalization, major cardiovascular events, and all-cause mortality (16–18, 44).

Despite converging evidence linking T2DM to both cognitive impairment and early cardiac dysfunction, the specific relationship between GLS and cognitive performance has not yet been examined in T2DM. Therefore, the aim of the present study was to investigate the association between GLS and cognitive performance in a well-characterized cohort of patients with T2DM.

## Materials and methods

### Study population

The study included individuals with T2DM who were consecutively and prospectively enrolled between July 2019 and February 2025 as part of the ongoing “Traguardi di Eccellenza nelle Scienze mediche Esplorando le Omiche” (TESEO) cohort, which investigates chronic complications of T2DM. Eligible participants were adults (≥18 years) referred for the first time to the Unified Diabetes Center at San Giovanni Antica Sede Hospital (Turin), who underwent baseline transthoracic echocardiography with speckle-tracking analysis. Exclusion criteria were: history of stroke, cognitive impairment interfering with daily functioning, major psychiatric disorders, internal carotid artery stenosis >70%, history of severe hypoglycemia, active malignancy, heart failure, inadequate acoustic window preventing GLS assessment, or inability to complete the RBANS due to patient refusal, language barriers, or significant visual impairment.

Upon enrollment, detailed demographic and clinical data were collected, including age, sex, ethnicity, educational level, dietary habits, physical activity, alcohol and tobacco use, cardiovascular risk factors, hypoglycemia history, comorbidities, chronic diabetes complications, and current pharmacologic treatments. Cognitive function was assessed using the Repeatable Battery for the Assessment of Neuropsychological Status (RBANS), administered by a single trained examiner to ensure consistency. All participants underwent a complete physical examination, fasting blood sampling for biochemical analyses, and morning urine collection to determine the albumin-to-creatinine ratio (ACR). Additional assessments included fundus oculi examination, 12-lead electrocardiogram (ECG), Doppler ultrasound of the supra-aortic trunks (carotid duplex), and transthoracic echocardiography.

**Figure 1:**
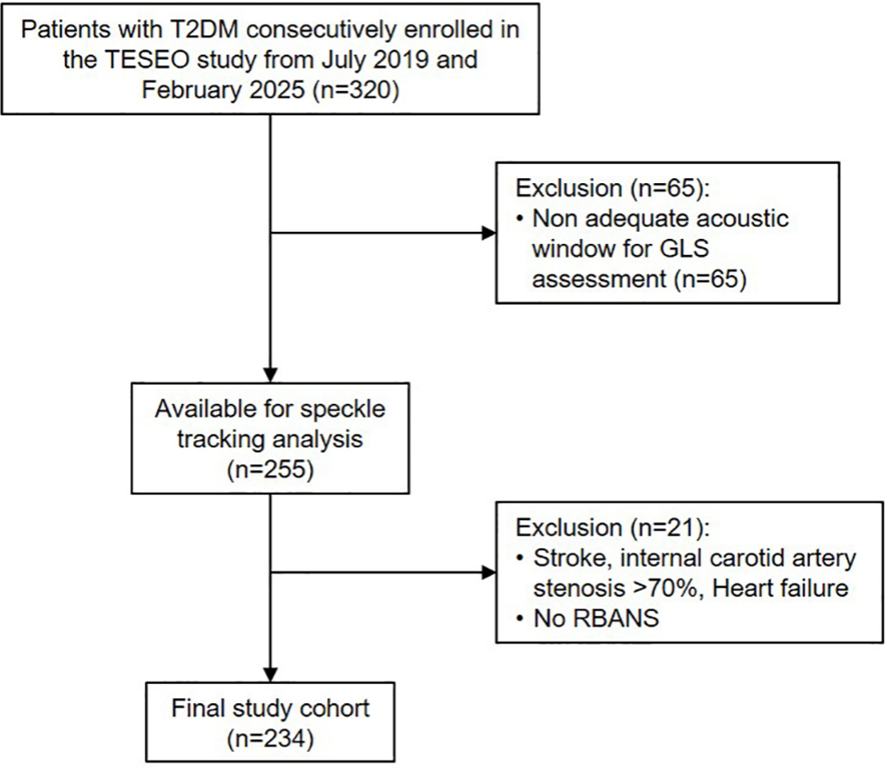
Flow chart of the study.

Of the 320 participants initially enrolled, 234 individuals were included in the final analysis (**Figure 1**). The study was approved by the Ethics Committee of the City of Health and Science of Turin, and all participants provided written informed consent.

### Biochemistry

Glycated hemoglobin (HbA1c) levels were determined using an immune-enzymatic method, and results were standardized according to the Diabetes Control and Complications Trial (DCCT) reference system. Plasma glucose, total cholesterol, triglycerides, high-density lipoprotein (HDL) cholesterol, and serum creatinine were measured using automated enzymatic assays on a Cobas-Bio analyzer. Urinary albumin and creatinine concentrations were assessed via an immunoturbidimetric technique. High sensitive C reactive protein (hs-CRP) was measured by immunoturbidimetry (Roche-Diagnostic).

### Definitions and calculations

Educational attainment was recorded on a 5-point ordinal scale: 0 = no formal education; 1 = primary school; 2 = middle school; 3 = high school or equivalent; 4 = university degree or higher. For the purposes of statistical analysis, this variable was dichotomized into *low educational attainment* (levels 0–2) and *high educational attainment* (levels 3–4). Physical activity levels were classified as low, moderate, or high based on responses to the short version of the International Physical Activity Questionnaire (IPAQ-short) (19). Sedentary behavior was quantified using the IPAQ-short item on daily sitting time. Participants were categorized into three groups based on smoking status: current smokers, former smokers, and never smokers. Body mass index (BMI) was calculated as weight in kilograms divided by height in meters squared (kg/m²). Waist circumference (WC) was measured in the horizontal plane at the upper edge of the right iliac crest, following a normal expiration. Visceral obesity was defined as a WC ≥94 cm in men and ≥80 cm in women (20). General obesity was defined as a BMI ≥30 kg/m². Blood pressure (BP) was measured using a standard manual sphygmomanometer (Hawksley, Lancing, UK). Hypertension was defined as systolic BP ≥130 mmHg and/or diastolic BP ≥80 mmHg, measured in the seated position after at least 5 minutes of rest with the left arm at heart level, confirmed on at least two separate visits, or current use of antihypertensive medications (21). Low-density lipoprotein cholesterol (LDL-C) was estimated using the Friedewald formula. Participants were considered albuminuric if their urinary albumin-to-creatinine ratio (ACR) was ≥3 mg/mmol in at least two of three samples collected within a six-month interval. Estimated glomerular filtration rate (eGFR) was calculated from serum creatinine using the CKD-EPI formula. Chronic kidney disease (CKD) was defined as eGFR ≤60 ml/min/1.73 m². Diabetic retinopathy was evaluated by a specialist ophthalmologist using retinal images acquired with the Optomed Aurora device (Midimedical). Retinopathy was classified as absent or present, with the latter subdivided into non-proliferative and proliferative forms. Non-proliferative retinopathy was diagnosed in the presence of microaneurysms, retinal hemorrhages, or hard exudates, while proliferative retinopathy was identified by neovascularization, fibrous tissue proliferation, or preretinal/vitreous hemorrhages. The more severely affected eye was used for classification. Coronary heart disease (CHD) was defined as a documented history of myocardial infarction, angina pectoris, percutaneous coronary intervention (PCI), or coronary artery bypass grafting (CABG). Participants showing clinical, electrocardiographic, or echocardiographic signs suggestive of CHD were classified as CHD patients if the diagnosis was confirmed by further evaluation.

### Echocardiography

Transthoracic echocardiography was performed using the EPIQ CVx ultrasound platform (Philips Healthcare, Andover, MA, USA) equipped with an X5–1 matrix-array transducer. All examinations were conducted by a cardiologist accredited by the European Association of Cardiovascular Imaging (EACVI), following standardized acquisition protocols to ensure consistency and reproducibility. High-resolution two-dimensional images were obtained from standard parasternal, apical, and subcostal views in accordance with EACVI guidelines. Frame rates were optimized between 50 and 80 frames per second to enhance temporal resolution for dynamic measurements. Left ventricular ejection fraction (EF) was quantified by two-dimensional biplane Simpson’s method from non-foreshortened apical four- and two-chamber views, with endocardial borders traced at end-diastole and end-systole. Measurements were averaged over three cardiac cycles (and five cycles in atrial fibrillation). Comprehensive Doppler imaging—including pulsed-wave, continuous-wave, color Doppler, and tissue Doppler imaging (TDI)—was utilized to assess valvular function and intracardiac flow dynamics. Doppler settings were customized based on individual hemodynamic characteristics. Image analysis and post-processing were carried out using QLab (Philips Healthcare) in combination with TomTec Arena (TomTec Imaging Systems). GLS of the left ventricle was measured using the AutoStrain feature, which facilitates semi-automated tracing of the endocardial borders. Manual corrections were applied as needed to improve tracking fidelity. GLS was calculated as the average of peak longitudinal strain values from the apical four-chamber, two-chamber, and three-chamber views. Segments with inadequate tracking were excluded based on visual inspection. A GLS value equal to or greater than −18% was classified as abnormal, reflecting early systolic dysfunction (14, 22). Additional structural and functional parameters were assessed, including LVEF, end-diastolic diameter (EDD), end-diastolic volume (EDV), end-systolic volume (ESV), interventricular septal (IVS) and posterior wall (PW) thickness, left ventricular (LV) mass, and relative wall thickness (RWT).

### Cognitive assessment

Cognitive function was evaluated using the RBANS, a standardized and validated tool widely used to detect and profile cognitive impairment in both clinical and research contexts (23). The RBANS includes 12 subtests grouped into five cognitive domains: Immediate Memory, assessed via List Learning (recall of a 10-word list over four trials) and Story Memory (recall of a short narrative); Visuospatial/Constructional Abilities, evaluated through Figure Copy (reproduction of a complex geometric figure) and Line Orientation (matching the angle and orientation of lines); Language, assessed using Picture Naming (naming familiar objects) and Semantic Fluency (generating words within a semantic category under time constraints); Attention, measured through Digit Span (repetition of number sequences both forward and backward) and Coding (a timed symbol-digit substitution task); Delayed Memory, evaluated using List Recall, List Recognition, Story Recall, and Figure Recall, reflecting retention of information from the Immediate Memory and Visuospatial tasks. Each domain yields a standardized index score, and a Total Scale Index Score (range 40-160) provides a composite measure of global cognitive functioning. Performance categories are defined as: Exceptionally High (≥130), High Average (110–129), Average (90–109), Low Average (80–89), Borderline (70–79), and Impaired (<70). The RBANS was administered by a trained examiner following standardized protocols in a quiet, controlled environment to minimize distractions. The total administration time ranged from approximately 20 to 30 minutes per participant.

### Statistical analysis

Continuous variables are expressed as mean ± standard deviation (SD) when normally distributed, and as geometric mean with interquartile range (25th–75th percentile) for non-normally distributed data (triglycerides, ACR, hs-CRP). Categorical variables are summarized as absolute frequencies and percentages. The normality of distributions was evaluated using both the Shapiro–Wilk and Kolmogorov–Smirnov tests. Between-group comparisons for continuous variables were conducted using two-tailed Student’s t-tests, while categorical variables were compared using the Chi-square test. In addition, RBANS scores were compared between participants with impaired GLS (≥−18%) and those without. Associations between GLS and RBANS total scores were first explored using univariate linear regression. Two separate multivariable linear regression models were constructed: one to assess the association between GLS and cognitive performance, and another to assess the association between EF and cognitive performance, each adjusted for relevant covariates (age, sex, education, sedentary behavior, smoking, HbA1c, systolic blood pressure, total cholesterol, eGFR). Additional analyses were conducted in a restricted subgroup excluding participants with CHD, atrial fibrillation (AF), or reduced EF (<50%). Furthermore, to assess whether the association between GLS and RBANS total score was influenced by the educational level, an interaction term between GLS and education was included in the regression model. All statistical tests were two-sided, and a p-value of <0.05 was considered statistically significant. Analyses were carried out using SPSS Statistics software, version 28.0 (IBM Corp., Armonk, NY, USA).

## Results

### Study population

**Table 1** presents the demographic, anthropometric, and clinical characteristics of the 234 individuals with T2DM included in the study. The mean age of participants was 61.6 ± 7.9 years, with a predominance of males. Regarding educational background, 59% had attained at least a high school diploma or university degree. Current smoking was reported by 22.6% of participants. Only a small proportion (13.5%) engaged in high levels of physical activity, while average daily sitting time was elevated, reflecting a predominantly sedentary lifestyle. Visceral obesity was present in 94.9% of the cohort.

**Table 1:** Demographic and clinical characteristics of the 234 subjects with type 2 diabetes of the TESEO study.

Variables	Study population n = 234
Age (yrs)	61.59 ± 7.86
Male gender (%)	63.2
Smoking status (%)	
No smokers	77.4
Active smokers	22.6
Education (%)	
No formal education	0
Primary school	7.3
Middle school	33.8
High school or equivalent	38.9
University degree or higher	20
Physical Activity (%)	
Low	35.2
Intermediate	51.3
High	13.5
Sedentary behavior (min/day)	279.13 ± 159.54
Body mass index (Kg/m^2^)	30.35 ± 5.37
Waist circumference (cm)	108.65 ± 11.71
Body mass Index ≥ 30 (%)	48.7
Diabetes duration (yrs)	3.86 ± 4.88
HbA1c (%)	6.53 ± 1.01
Systolic BP	135.19 ± 16.19
Diastolic BP	82.27 ± 9.92
Total cholesterol (mg/dl)	170.11 ± 41.36
LDL-cholesterol (mg/dl)	94.36 ± 35.52
HDL-cholesterol (mg/dl)	50.36 ± 12.23
Triglycerides (mg/dl)	114.41 (87.0-147.0)
ACR (mg/mmol)	1.05 (0.60-1.58)
eGFR (ml/min/1.73m^2^)	86.60 ± 16.14
AF (%)	2.6
LVEF (%)	
≥50	96.6
49-41	3.0
≤40	0.4
Uric Acid (mg/dl)	5.54 ± 1.60
hs-CRP (mg/L)	2.11 (0.98 - 4.08)

Data are expressed as mean ± SD, percentage or geometric mean (25°-75° percentile) for log transformed data (triglycerides, ACR, hs-CRP). BP, blood pressure; ACR, albumin-creatinine ratio; eGFR, estimated glomerular filtration rate; LDL, low-density lipoprotein; HDL, high-density lipoprotein; AF, atrial fibrillation; LVEF, left ventricular ejection fraction; hs-CRP, high-sensitivity C-reactive protein.

Overall, the participants had a relatively short duration of T2DM and exhibited good glycemic control. A small proportion (8.1%) were on medications known to increase the risk of hypoglycemia, and no participant reported prior hypoglycemic episodes. The prevalence of comorbidities and diabetes-related chronic complications was as follows: hypertension in 91.5%, albuminuria in 13.7%, CKD in 9.4%, diabetic retinopathy in 4.8%, and CHD in 12.4%.

### GLS and RBANS

In the overall study population, the mean RBANS Total Scale Index Score was 96.70 ± 17.10 (**Table 2**). A total of 19.7% of participants scored below 80 on the RBANS total scale, indicating borderline/impaired cognitive performance.

**Table 2:** RBANS index scores of the 234 subjects with type 2 diabetes of the TESEO study.

RBANS domain index scores	*n =* 234
Immediate Memory	93.08 ± 15.42
Visuospatial/Constructional	102.33 ± 18.73
Language	90.17 ± 11.28
Attention	103.25 ± 18.82
Delayed Memory	101.80 ± 16.49
Total score	96.70 ± 17.10

Data are expressed as mean ± SD.

GLS values were normally distributed, with a mean of −19.23 ± 2.59. Notably, 29.1% of participants had GLS values ≥ −18%, consistent with subclinical left ventricular systolic dysfunction. Participants with abnormal GLS values (≥ −18%) demonstrated significantly lower RBANS total scores compared to those with normal GLS (92.97 ± 16.58 vs. 98.23 ± 17.14; p = 0.032).

Univariate linear regression analysis revealed a significant association between GLS and RBANS total score [β = -0.196 (-0.323; -0.069); p = 0.003]. When individual cognitive domains were examined, GLS was significantly associated with Immediate Memory [β = -0.198 (-0.325; -0.071), p = 0.002], Attention [β = -0.251 (-0.376; -0.126), p < 0.001], and Language [β = -0.163 (-0.291; -0.035), p = 0.013]. No significant associations were found for Visuospatial/Constructional abilities [β = -0.049 (-0.178; 0.080), p = 0.455] or Delayed Memory [β = -0.092 (-0.220; 0.036), p = 0.159].

### Multivariate linear regression analyses: GLS and cognitive performance

Multivariate linear regression analyses were performed to evaluate whether GLS was independently associated with the RBANS total score after adjusting for potential confounders. As shown in **Table 3**, GLS remained significantly associated with the RBANS total score [β = -0.174 (-0.285; -0.063), p = 0.002], after controlling for age, sex, education level, sedentary behavior, smoking status, HbA1c, systolic blood pressure, total cholesterol, and eGFR. This relationship was primarily driven by significant independent associations between GLS and the Immediate Memory, Attention, and Language domains.

**Table 3:** Associations between cardiac dysfunction and RBANS total and single scores.

	All cohort		Patients without CHD, AF, EF<50%	
	n=234		n=196	
	β (95% CI)	P value	β (95% CI)	P value
RBANS Total Score				
GLS	-0.174 (-0.285; -0.063)	**0.002**	-0.195 (-0.313; -0.077)	**0.001**
LVEF	0.106 (-0.036; 0.787)	0.073	0.052 (-0.071; 0.175)	0.407
RBANS Immediate Memory				
GLS	-0.183 (-0.302; -0.064)	**0.003**	-0.153 (-0.280; -0.026)	**0.020**
LVEF	0.089 (-0.113; 0.684)	0.159	0.035 (-0.096; 0.166)	0.603
RBANS Visual				
GLS	-0.044 (-0.162; 0.074)	0.461	-0.105 (-0.233; 0.023)	0.112
LVEF	0.020 (-0.101; 0.141)	0.747	0.000 (-0.079; 0.079)	1.000
RBANS Attention				
GLS	-0.249 (-0.363; -0.134)	**<0.001**	-0.204 (-0.328; -0.080)	**0.002**
LVEF	0.159 (0.038; 0.280)	**0.011**	0.097 (0.032; 0.226)	0.144
RBANS Delayed Memory				
GLS	-0.072 (-0.190; 0.046)	0.233	-0.130 (-0.256; -0.044)	**0.044**
LVEF	0.017 (-0.106; 0.140)	0.787	0.000 (-0.114; 0.114)	0.998
RBANS Language				
GLS	-0.132 (-0.253; -0.011)	**0.034**	-0.104 (-0.236; 0.028)	0.125
LVEF	0.089 (-0.037; 0.215)	0.169	0.020 (-0.114; 0.154)	0.768

Models were adjusted for age, sex, education, sedentary behavior, smoking, HbA1c, systolic blood pressure, total cholesterol, eGFR. CI, confidence interval; RBANS, Repeatable Battery for the Assessment of Neuropsychological Status; GLS, global longitudinal strain; LVEF, left ventricular ejection fraction; CHD, coronary heart disease; AF, atrial fibrillation; EF, ejection fraction. *Bold significant p-values.

Sensitivity analyses, excluding participants with prevalent CHD, atrial fibrillation (AF), and/or LVEF below 50%, were conducted in a subgroup of 196 individuals. In these patients, GLS remained significantly and independently associated with the RBANS total score [β = -0.195 (-0.313; -0.077), p = 0.001], as well as with Immediate Memory, Delayed Memory, and Attention domains. In addition to GLS, other variables significantly associated with the RBANS total score included education [β = 0.480 (0.354; 0.606), p < 0.001], eGFR [β = -0.145 (-0.286; -0.004), p = 0.045], and sedentary behavior [β = 0.129 (0.003; 0.255), p = 0.046]. Notably, a significant interaction was observed between education (five levels) and GLS in predicting the RBANS total score [β = -0.517 (-0.759; -0.275), p < 0.001], suggesting that the association between cardiac function and cognitive performance varies according to educational attainment.

Importantly, in the subgroup of 196 patients without CHD, AF, or LVEF <50%, the association between GLS and the RBANS total score remained significant after additional adjustment for ln-ACR (β = -0.192 (-0.313; -0.071), p = 0.002]. Furthermore, in a subset of 181 patients with available hs-CRP measurements, adjustment for both ln-ACR and ln-CRP did not alter the results [β = -0.191 (-0.317; -0.065), p = 0.003]. Similarly, the results remained unchanged after adjustment for ACE inhibitor/angiotensin II receptor blocker treatment [β = –0.187 (-0.31; -0.07)] or for lipid-lowering medications [β = -0.196 (-0.314; -0.078)].

### Multivariate analysis: LVEF and cognitive performance

Additional multivariate linear regression analyses were conducted using LVEF as the predictor variable. In the fully adjusted model, LVEF was not independently associated with the RBANS total score. A significant relationship emerged only with the Attention domain; however, this association was weaker than those observed with GLS.

When the analysis was restricted to participants without CHD, atrial fibrillation, or reduced LVEF (<50%), no significant associations were found between LVEF and the RBANS total score or any of the individual cognitive domains.

## Discussion

Our study demonstrates a strong and independent association between impaired GLS and reduced cognitive performance in individuals with T2DM, who had no history of stroke, severe hypoglycemic episodes, or hemodynamically significant carotid artery stenosis. Importantly, this relationship persisted after excluding patients with CHD, atrial fibrillation, and reduced EF, and adjusting for a wide range of potential confounders, including age, sex, education, lifestyle factors, metabolic control, blood pressure, lipid profile, and kidney function. In contrast, LVEF showed no independent association with cognitive performance, underscoring the superior sensitivity of GLS as an early marker of subclinical cardiac dysfunction with cognitive implications.

These findings are consistent with previous studies in non-diabetic populations. The Vanderbilt Memory and Aging Project reported that subclinical LV dysfunction, measured by GLS on cardiac MRI, was associated with poorer episodic memory and language skills, despite preserved LVEF (24). Similarly, in hypertensive individuals with normal EF, GLS was linked to the presence of mild cognitive impairment (25). Moreover, community-based data have shown that GLS, but not LVEF, is significantly associated with silent brain infarcts and white matter hyperintensities (26).

Cognitive performance in our study was evaluated using the RBANS, a validated, domain-specific tool that allows for comprehensive evaluation of cognitive functioning. Unlike brief global assessments, such as the Mini-mental State Examination (MMSE) or Montreal Cognitive Assessment (MoCA), RBANS provides a multidimensional evaluation across five cognitive domains, enabling the identification of selective impairments (27). Moreover, RBANS sensitivity to mild cognitive deficits makes it particularly suited for detecting early cognitive changes in populations at elevated risk, such as patients with T2DM. In our cohort, 20% of participants scored below the RBANS cut-off of 80, indicating borderline/impaired cognitive performance. While estimates of cognitive impairment prevalence in T2DM vary widely due to differences in study populations and assessment tools (28), our results align with previous studies using similar cognitive measures (29). Domain-specific analyses revealed that GLS was most strongly associated with Immediate Memory, Attention, and Language - functions typically affected in the early stages of diabetes-related cognitive impairment. This selective pattern suggests that subclinical LV systolic dysfunction may influence specific neural networks.

The mechanisms underlying the link between subclinical cardiac dysfunction and cognitive impairment remain incompletely understood but are likely multifactorial. While diabetes-related factors such as chronic hyperglycemia, hypertension, and dyslipidemia contribute to cognitive decline (30, 31), our findings indicate that the association between GLS and cognition persists independently of these traditional risk factors.

Moreover, further adjustment for ACE inhibitor/angiotensin II receptor blocker treatment or lipid lowering medications did not modify the results. One hypothesis is that microvascular dysfunction, driven by endothelial damage, oxidative stress, and inflammation, may simultaneously affect cerebral and myocardial microcirculation (32) In this context, impaired GLS may reflect microangiopathy affecting both organs. However, the observed associations remained significant even after adjusting for urinary ACR, a marker of renal microvascular disease and endothelial dysfunction, as well as for C-reactive protein, an inflammatory marker, making this explanation less likely. An alternative hypothesis is that subtle impairments in LV systolic function may compromise cardiac output and cerebral autoregulation, resulting in cerebral hypoperfusion (33–36). Chronic reductions in cerebral blood flow can lead to white matter lesions, cortical thinning, and ultimately, cognitive decline (37). This hemodynamic mechanism may provide a plausible link between subclinical cardiac dysfunction and cognitive performance.

In our cohort, 59% of participants had completed high school or university, and educational attainment emerged as the strongest predictor of RBANS scores in multivariate analyses. This finding is consistent with prior studies in both the general population and individuals with T2DM (38, 39). Education is widely recognized as a key contributor to cognitive reserve - the brain’s capacity to maintain cognitive function in the presence of pathology (40). In line with this, we observed a significant interaction between education and GLS in predicting RBANS total score, indicating that educational attainment is an effect modifier in the cardiac–cognitive relationship. This results in a more pronounced negative impact of impaired GLS on cognition in individuals with lower education levels.

From a clinical perspective, these findings carry important implications. Incorporating GLS measurement into routine echocardiographic evaluations for patients with T2DM could help identify individuals at elevated risk for cognitive decline. Early recognition of subclinical cardiac dysfunction may prompt timely, integrative interventions targeting both cardiovascular and cognitive health. The availability of cardioprotective therapies, including renin–angiotensin system inhibitors, sodium-glucose cotransporter 2 (SGLT2) inhibitors, and non-steroidal mineralocorticoid receptor antagonists (MRAs) (41, 43) opens the possibility for therapeutic strategies that may also preserve cognitive function. Future studies are warranted to explore whether improving cardiac function through such treatments confers neuroprotective benefits.

This study has several limitations. Its cross-sectional design precludes conclusions about causality; longitudinal research is necessary to determine whether impaired GLS predicts future cognitive decline. Second, although RBANS offers comprehensive cognitive coverage, inclusion of more specific assessments (e.g., executive function, processing speed) and neuroimaging data would provide deeper insights into the neural substrates involved. Third, recruitment from a single tertiary care center may limit the generalizability of our findings to broader T2DM populations. Nevertheless, our study has notable strengths, including a relatively large sample size, use of standardized neuropsychological testing, real-time echocardiographic interpretation by a cardiologist certified by the EACVI, and robust adjustment for a wide range of confounders. In addition, carotid ultrasound was performed in all participants, allowing exclusion of significant stenosis, an important advantage over reliance on self-reported history. Finally, inclusion of microvascular, inflammatory, and endothelial markers, allowed us to explore potential underlying mechanisms.

In conclusion, this is the first study to demonstrate that subclinical LV systolic dysfunction, as identified by impaired GLS, is independently associated with specific cognitive deficits in patients with T2DM, beyond what is captured by conventional LVEF measurements. These findings highlight the potential of GLS as a dual-purpose biomarker for identifying individuals at increased cardiac and cognitive risk, and support its integration into future prospective studies and multidisciplinary diabetes care models.

## Data Availability

The raw data supporting the conclusions of this article will be made available by the authors, without undue reservation.

## Glossary

ACR: albumin-to-creatinine ratio
BMI: Body mass index
BP: Blood pressure
CABG: coronary artery bypass grafting
CHD: Coronary heart disease
CKD: Chronic kidney disease
DCCT: Diabetes Control and Complications Trial
eGFR: Estimated glomerular filtration rate
EDV: end-diastolic volume
EDD: end-diastolic diameter
EACVI: European Association of Cardiovascular Imaging
ECG: electrocardiogram
ESV: end-systolic volume
GLS: Global Longitudinal Strain
HbA1c: Glycated hemoglobin
HDL: high-density lipoprotein
HF: heart failure
hs-CRP: High sensitive C reactive protein
IPAQ-short: International Physical Activity Questionnaire (short form)
IVS: interventricular septal thickness
LVEF: left ventricular ejection fraction
LDL-C: Low-density lipoprotein cholesterol
LV mass: left ventricular mass
MRAs: non-steroidal mineralocorticoid receptor antagonists
PCI: percutaneous coronary intervention
PW: posterior wall thickness
RBANS: Repeatable Battery for the Assessment of Neuropsychological Status
RWT: relative wall thickness
SGLT2: sodium-glucose cotransporter 2 inhibitors
TDI: tissue Doppler imaging
T2DM: Type 2 diabetes mellitus
WC: Waist circumference
